# The association between psoriatic arthritis and venous thromboembolism: a population-based cohort study

**DOI:** 10.1186/s13075-021-02703-8

**Published:** 2022-01-07

**Authors:** Tal Gazitt, Jacob Pesachov, Idit Lavi, Muna Elias, Amir Haddad, Ilan Feldhamer, Arnon Dov Cohen, Walid Saliba, Devy Zisman

**Affiliations:** 1 Rheumatology Unit, Carmel Medical Center, Michal 7 St, 3436212 Haifa, Israel; 2 Department of Medicine, Division of Rheumatology, University of Washington Medical Center, Seattle, Washington USA; 3 The Ruth and Bruce Rappaport Faculty of Medicine, Technion, Haifa, Israel; 4 Department of Community Medicine and Epidemiology, Carmel Medical Center, Haifa, Israel; 5 Central Headquarters, Clalit Health Services, Tel Aviv, Israel; 6 Siaal Research Center for Family Medicine and Primary Care, Faculty of Health Sciences, Ben-Gurion University of the Negev, Beer-Sheva, Israel

**Keywords:** psoriatic arthritis, psoriasis, risk factors, spondyloarthropathy, venous thromboembolism

## Abstract

**Background:**

Although the risk of cardiovascular disease has been discussed extensively in both psoriasis (PsO) and psoriatic arthritis (PsA), very few studies have addressed the occurrence of venous thromboembolic (VTE) events among PsO patients, and even fewer in PsA. Thus, our goal was to assess the association between PsA and VTE events using a large population-based database.

**Methods:**

This retrospective cohort study includes all 5,275 patients with newly diagnosed PsA from the largest health care provider in Israel between January 2003 and December 2018. Identified PsA patients were matched by age, sex, ethnicity, and index date with 21,011 controls without PsA from the same database. Both groups were followed through June 30, 2019 for the occurrence of VTE event. Cox proportional hazard regression models were used to assess the association between PsA and VTE.

**Results:**

PsA cohort consisted of 53.2% females with mean age of 51.7±15.4 Sixty-two patients (1.2%) were diagnosed with VTE in the PsA group and 176 patients (0.8%) in the control group (p=0.023, HR=1.40, 95% CI 1.05-1.87). However, there was no increased risk of VTE among PsA patients on multivariable analysis (p=0.16, HR=1.27, 95% CI 0.91-1.80). Within the PsA group, patients with VTE were more often of older age and with history of VTE.

**Conclusions:**

This study suggests that the increased risk of VTE in PsA patients appears to be related to the underlying comorbidities and not independently associated with PsA. Age and previous history of VTE were the only risk factors associated with increased risk of VTE in patients with PsA. Addressing VTE risk is recommended especially in the era of Janus kinase inhibitors.

## Introduction

Psoriatic arthritis (PsA) belongs to the seronegative spondyloarthropathies, a group of rheumatic diseases that have common genetic associations and share certain clinical features aside from peripheral arthritis, such as spondylitis, enthesitis, dactylitis, uveitis, and inflammatory bowel disease. PsA has been found to be associated with several comorbidities including obesity, diabetes, hypertension, hyperlipidemia, fatty liver disease, osteoporosis, and cardiovascular disease (CVD) including ischemic heart disease (IHD), congestive heart failure (CHF), peripheral vascular disease (PVD), cardiomyopathy, and valvular heart disease [1–3].

Venous thromboembolic events (VTE) comprise of two primary forms: occurrence of deep-vein thrombosis (DVT) in the extremities and any subsequent embolization to the lungs, termed pulmonary emboli (PE). It is estimated that 300,000–600,000 new cases of venous thromboembolism occur each year in the United States alone with 60,000–80,000 deaths attributed to DVT or PE [4].

Although the risk of cardiovascular disease has been discussed extensively in both psoriasis (PsO) and PsA, very few studies have addressed the occurrence of VTE events among PsO patients, [5, 6] and even fewer in PsA. This is of particular significance given the recent concern raised over increased incidence of VTE events among patients with rheumatoid arthritis (RA), PsO, and PsA with pre-existing cardiovascular risk factors treated with tofacitinib [7] and initially reported by the Data Safety Monitoring Board of the ongoing, post-authorization safety surveillance study A3921133 (NCT02092467) in RA patients aged ≥50 years and with ≥1 cardiovascular risk factor treated by tofacitinib 10 mg twice daily dose. Thus, our goal was to evaluate the incidence of VTE in a cohort of PsA relative to a comparator group without PsA using a large population-based database.

## Materials and methods

### Source of data

This study is based on the computerized database of Clalit Health Services (CHS), which is the largest healthcare provider in Israel, serving approximately 4.7 million members constituting ~52% of Israel’s population. The CHS membership is comprised of individuals of widely diverse geographic distribution, different ethnicities, and from all socioeconomic backgrounds across Israel, with all members having equal access to the same uniform medical benefits granted by the Israeli National Healthcare Plan, as mandated by the Israeli National Health Inusrance Law (1995). According to this law, each of the 4 major healthcare providers in Israel, one of which is CHS, are akin to not-for-profit Health Maintenance Organizations (HMOs) which serve as both healthcare insurers and providers, thus financing and providing medical services to their members. Membership in each of the 4 HMOs is voluntary and members can freely move from one healthcare insurance organization to another. All 4 healthcare organizations provide similar healthcare insurance plans and provide similar access to health services and medical benefits, including low medication copayments, as stipulated by the Israeli National Health Insurance Law and the Israeli National Healthcare Plan which is updated on an annual basis.

CHS maintains a database that receives information updated continuously from pharmaceutical, medical and administrative operating systems. Disease codes are registered according to the International Classification of Diseases 9th Revision (ICD-9) and medications dispensed are coded according to the Anatomical Therapeutic Chemical (ATC) classification. The database was designed for purposes of administrative and clinical management and is available for use in epidemiological studies. A registry of chronic diseases diagnoses is compiled from the different data sources. Diagnoses are captured in the registry by diagnosis-specific algorithms, employing ICD-9 code reading, text reading, laboratory test results and disease-specific drug usage. The validity of selected disease diagnoses in the CHS database was found to be high in previous studies [8], and specifically the algorithm used to retrieve PsA patients has been previously validated by our group and found to have high sensitivity (88.7%), specificity (88.1%), and positive predictive value (90.5%) [9].

### Study population

The selection of the population of this retrospective cohort study was previously described elsewhere [10]. Briefly, the CHS database was interrogated for adult patients who were newly diagnosed with PsA between January 1, 2003 (start date) and until December 31, 2018 with date of diagnosis considered the index date. A risk set sampling was employed to randomly select up to 4 controls without PsA as comparator group for each PsA patient included in the study with controls matched by age (within 1 year), sex, ethnic group (Jews and Arabs), and index date starting January 1, 2003. Both groups were followed from the index date until the first occurrence of hospital-based diagnosis of VTE event, death, or end of follow-up June 30, 2019 (end date), whichever came first, so that only VTE events occurring following diagnosis of PsA were included in the analysis.

### Study variables

The study outcome was defined as a primary discharge diagnosis with VTE consisting of the composite of deep venous thrombosis (ICD-9, 453.4X, 451.1X) and pulmonary embolism (ICD-9, 415.1X). In addition, for each patient, the following data were retrieved from the CHS database: demographic variables, smoking status, socioeconomic status (SES) based on the SES score assigned to clinic neighborhoods as defined by the Israeli Central Bureau of Statistics, body mass index (BMI), presence of selected chronic comorbidities, and medication use including conventional and biologic disease-modifying anti-rheumatic drugs (c/b DMARDs).

### Statistical analysis

Continuous variables were summarized as mean ± standard deviation (SD), while categorical variables were presented as numbers and proportions. Comparisons of baseline characteristics between patients with and without PsA were done using chi-square test for categorical variables and using Student's t-test for continuous variables, as appropriate. In addition, baseline differences among the PsA patient group and controls were calculated using standardized mean difference (SMD) [11] with SMD ≤0.1 indicating a negligible difference in the measured variables between groups.

**Fig. 1:**
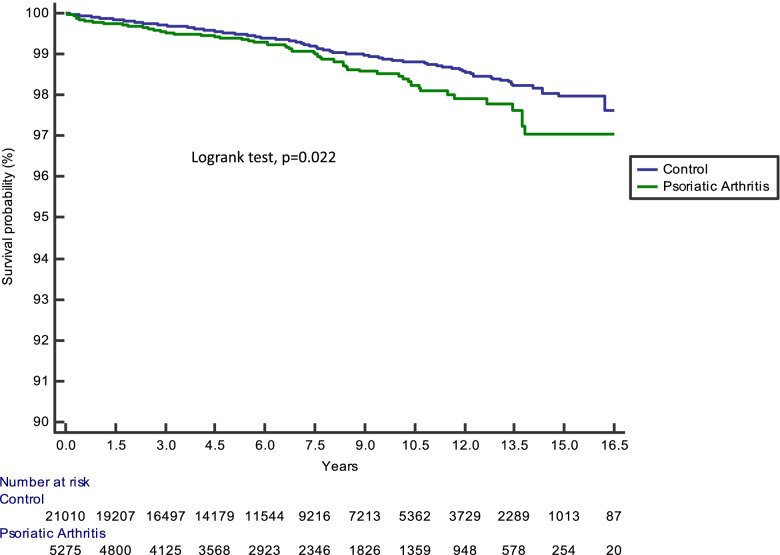
Kaplan-Meier curve showing accrual of VTE events over time in PsA group vs control group

Kaplan-Meier curves were used to plot the distribution of time to reach VTE events in PsA patients and controls, and the curves were compared by log-rank test (Fig. 1). Cox proportional hazard regression models were used to estimate the crude hazard ratios (HRs) (univariable analysis; because of matching by age, sex, ethnicity, and index date, this analysis should be considered as adjusted for these variables) and adjusted HR (multivariable analysis; controlling for demographic variables, SES, smoking, and various comorbidities, including obesity, CVD risk factors, and vascular disease) for the association between PsA and VTE. We also analyzed VTE occurrence in the PsA patient group. In this last analysis, Cox proportional time-dependent models were used to estimate the HR for the association between c/bDMARD and VTE. We then performed a sensitivity analysis in which we limited analysis of VTE occurrence to individuals without previous history of VTE events in both PsA patients and controls.

All data were analyzed using SPSS, 24 (IBM Corp. Released 2016. IBM SPSS Statistics for Windows, version 24.0, 2016, Armonk, NY) and SAS, 9.4 (SAS institute Inc, Cary, NC). In all analyses, P≤0.05 for the 2-tailed tests was considered statistically significant.

The study was approved by the Institutional Review Board of Carmel Medical Center (CMC-0014-14). Requirement for individual patient consent forms was waived due to the retrospective, observational nature of the study.

## Results

The PsA cohort consisted of 5,275 patients, 53.2% of whom were females with mean age of 51.7 ±15.4 (Table 1). The control group consisted of 21,011 subjects matched for age, sex, ethnicity, and index date. Compared to the control group, the PsA cohort had more psoriasis (80.5% vs 1.6%, p<0.0001, SMD 2.7) and higher prevalence of cardiovascular disease-associated risk factors such as diabetes (33.8% vs 26.2%, p<0.0001, SMD=0.2), and obesity (BMI≥30 in 33.5% vs 25.8%, p<0.0001, SMD=0.2) (Table 1).

**Table 1:** Baseline characteristics of the study population

		PsA	Control	*p*-value	SMD^b^
Number of patients		5275	21011		
Age (Mean± SD, Median)		51.7 ±15.4, 52.6	51.7 ± 15.5, 52.6	0.99	0.00
Sex	Female	2807 (53.2%)	11173 (53.2%)	0.96	0.00
Ethnicity	Jewish	4594 (87.1%)	18296 (87.1%)	0.98	
	Arab	681 (12.9%)	2715 (12.9%)		0.00
Socio-economic status^a^	1-low	1621 (30.7%)	7169 (34.1%)	<0.0001	0.04
	2-medium	2043 (38.7%)	8779 (41.8%)		
	3-high	1326 (25.1%)	4922 (23.4%)		
Tobacco use		2227 (42.2%)	8311 (39.6%)	<0.0001	0.05
BMI^a^	BMI<25	1480 (28.0%)	7760 (36.9%)	<0.0001	0.2
	BMI≧25<30	1795 (34.0%)	7546 (35.9%)		
	BMI≧30	1765 (33.5%)	5411 (25.8%)		
Psoriasis		4244 (80.5%)	337 (1.6%)	<0.0001	2.7
Comorbidity	Cancer	337 (6.4%)	1284 (6.1%)	0.45	0.01
	Diabetes	1781 (33.8%)	5497 (26.2%)	<0.0001	0.2
	IHD	542 (10.3%)	1816 (8.6%)	<0.0001	0.06
	CVA/TIA	243 (4.6%)	824 (3.9%)	0.024	0.03
	CHF	117 (2.2%)	344 (1.6%)	0.004	0.04
	Hypertension	1589 (30.1%)	5495 (26.2%)	<0.0001	0.08
	AF	159 (3.0%)	520 (2.5%)	0.027	0.03
	Vascular Disease	196 (3.7%)	624 (3.0%)	0.005	0.04
	Past VTE	39 (0.7%)	109 (0.5%)	0.056	0.08
cDMARD		2053 (38.9%)	0 (0.0%)	<0.0001	1.1
bDMARD		2015 (38.2%)	107 (0.5%)	<0.0001	0.9

^a^Presented data for percentages for socioeconomic status and BMI were calculated from the total number of the available data. Missing data on socioeconomic status was 285 (5.4%) and 141 (0.7%) for PsA and control groups, respectively; missing data on BMI was 235 (4.5%) and 294 (1.4%) for PsA and control groups, respectively.

^b^Standardized mean difference (SMD) of ≤0.1 indicates a negligible difference in the measured variables between groups.

*Abbreviations*: *AF* Atrial Fibrillation, b/c DMARD=biologic/conventional disease-modifying anti-rheumatic drugs, *BMI* body mass index, *CHF* Congestive Heart Failure, *COPD* Chronic obstructive pulmonary disease, *CRF* Chronic renal failure, *CVA* Cerebrovascular accident, *IHD* Ischemic heart disease, *PsA* Psoriatic arthritis, *SD* Standard deviation, *TIA* Transient ischemic attack.

In general, the mean age of patients diagnosed with VTE was 64.9±13.2 years (Table 2). Notably, patients diagnosed with VTE were older than patients who did not experience VTE events (64.9±13.2 vs 51.5±15.4 years, P<0.0001), and these patients also suffered from more comorbidities which were found to be statistically significant in both univariate and multivariate analysis, such as cancer (2.2% vs 0.8%, p<0.0001), IHD (2.7% vs 0.7%, p<0.0001), vascular disease (3.5% vs 0.8%, p<0.0001) and past VTE (14.9% vs 0.8%, p<0.0001) (Table 2).

**Table 2:** Risk Factors for VTE

			Univariate Analysis			Multivariate analysis		
		+VTE	***P***. Value	HR	95% CI	***P***. Value	HR	95% CI
**PsA**	yes	62/5275 (1.2%)	0.023	1.40 Ref.	1.05-1.87	0.16	1.27	0.91-1.80
	no	176/21011 (0.8%)						
**Age** **(Mean± SD)**		64.9±13.2 (Without VTE 51.5 ±15.4)	<0.0001	1.08	1.07-1.10	<0.0001	1.08	1.06-1.10
**Sex**	Female	138/13980 (1.0%)	0.11	1.24	0.95-1.61	0.088	1.27	0.97-1.67
**Socio-economic status**^a^	1-low	72/8790 (0.8%)		Ref.			Ref.	
	2-medium	103/10822 (1.0%)	0.51	1.11	0.82-1.50	0.65	0.93	0.69-1.26
	3-high	57/6248 (0.9%)	0.74	1.06	0.75-1.50	0.58	0.91	0.64-1.290
**BMI**^a^	BMI<25	51/9240 (0.6%)		Ref.			Ref.	
	BMI≧25<30	87/9341 (0.9%)	0.0063	1.62	1.15-2.29	0.34	1.19	0.84-1.68
	BMI≧30	96/7176 (1.3%)	<0.0001	2.38	1.69-3.34	0.0068	1.67	1.15-2.42
**Tobacco use**	Yes	99/10538 (0.9%)	0.26	1.17	0.89-1.55	0.23	1.20	0.89-1.61
	No	139/15748 (0.9%)		Ref.			Ref	
**Comorbidity**	Cancer							
	yes	35/1621 (2.2%)	<0.0001	3.27	2.28-4.69	0.030	1.51	1.04-2.20
	No	203/24665 (0.8%)		Ref.			Ref.	
	Diabetes							
	Yes	91/7278(1.3%)	<0.0001	2.12	1.63-2.76	0.48	0.89	0.66-1.22
	No	147/19008 (0.8%)		Ref.			Ref.	
	IHD							
	Yes	64/2358 (2.7%)	<0.0001	4.40	3.30-5.87	0.018	1.52	1.07-2.14
	No	174/ 23928(0.7%)		Ref.			Ref.	
	CVA/TIA							
	Yes	27 (2.5%)	<0.0001	4.07	2.72-6.08	0.15	1.38	0.89-2.13
	No	211/25219 (0.8%)		Ref.			Ref.	
	CHF							
	Yes	16/461 (3.5%)	<0.0001	6.95	4.17-11.57	0.12	1.58	0.89-2.80
	No	222/25825 (0.9%)		Ref.			Ref.	
	Hypertension							
	Yes	118/7084 (1.7%)	<0.0001	2.91	2.26-3.75	0.23	0.83	0.61-1.13
	No	120/19202 (0.6%)		Ref.			Ref.	
	AF							
	Yes	18/379(2.7%)	<0.0001	4.33	2.67-7.01	0.76	0.92	0.54-1.56
	No	220/25607 (0.9%)		Ref.			Ref.	
	Vascular Disease							
	Yes	29/820 (3.5%)	<0.0001	5.85	3.96-8.64	0.020	1.66	1.08-2.55
	No	209/25466 (0.8%)		Ref.			Ref.	
	COPD							
	Yes	24/1885 (1.3%)	0.021	1.64	1.08-2.51	0.64	1.11	0.72-1.70
	No	214/24401 (0.9%)		Ref.			Ref.	
	CRF							
	Yes	15/688 (2.2%)	<0.0001	3.93	2.32-6.64	0.50	1.21	0.69-2.10
	No	223/25598 (0.9%)		Ref.			Ref.	
	Past VTE							
	Yes	22/148 (14.9%)	<0.0001	27.15	17.48-	<0.0001	13.00	8.21-20.56
	No	216/26138 (0.8%)		Ref.	42.18			

^a^Presented data for percentages for socioeconomic status and BMI were calculated from the total number of the available data. Missing data on socioeconomic status was 6/426 (1.4%) and for BMI was 4/529 (0.8%) for PsA+controls with VTE, respectively.

*Abbreviations*: *AF* Atrial fibrillation, *bDMARD* biologic disease-modifying anti-rheumatic drug, *BMI* body mass index, *CHF* Congestive heart failure, *CI* Confidence interval, *COPD* Chronic obstructive pulmonary disease, *CRF* Chronic renal failure, *CVA* cerebrovascular accident, *HR-* Hazard ratio, *IHD* Ischemic heart disease, *PsA* Psoriatic arthritis, *Ref.* Reference for calculation, *SD* Standard deviation, *TIA* Transient ischemic attack, *VTE* Venous thromboembolism.

During follow-up, there were 62 patients (1.2%) diagnosed with VTE in the PsA group vs 176 patients (0.8%) in the control group [p=0.023, crude HR=1.40 (95%CI 1.05-1.87)]. On multivariate analysis, following adjustment for multiple covariates including demographic variables, SES, smoking, and various comorbidities, including obesity, CVD risk factors, and vascular disease, this association did not remain statistically significant [p=0.16, adjusted HR=1.27 (95% CI 0.91-1.80)] (Table 2).

In the sensitivity analysis which excluded PsA patients and controls with previous history of VTE, 5,181 PsA patients and 20,590 controls were included. In this analysis, 55 PsA patients and 160 controls had VTE events [p=0.051, HR 1.36, CI (1.00-1.84) in univariate analysis; p=0.15, HR 1.26, CI (0.92-1.72) in multivariate analysis] (Table 3).

**Table 3:** Risk Factors for VTE excluding cases and controls with previous VTE

			Univariate Analysis			Multivariate analysis		
		+VTE	P. Value	HR	95% CI	P. Value	HR	95% CI
**PsA**	yes	55/5236 (1.1%)	0.051	1.36 Ref.	1.00-1.84	0.15	1.26	0.92-1.72
	no	160/20750 (0.8%)						
**Age (Mean± SD)**		64.60±13.04 (Without VTE 51.44 ±15.40)	<0.0001	1.08	1.07-1.09	<0.0001	1.07	1.06-1.08
**Sex**	Female	126/13808 (0.9%)	0.081	1.27	0.97-1.67	0.074	1.30	0.97-1.74
**Socio-economic status**^a^	1-low	62/8672(0.7%)		Ref.			Ref.	
	2-medium	95/10707 (0.9%)	0.307	1.18	0.86-1.63	0.76	0.95	0.69-1.31
	3-high	53/6184(0.9%)	0.472	1.14	0.79-1.65	0.86	0.97	0.67-1.40
**BMI**^a^	BMI<25	47/9166 (0.5%)		Ref.			Ref.	
	BMI≧25<30	79/9238(0.9%)	0.011	1.60	1.11-2.29	0.27	1.23	0.85-1.77
	BMI≧30	86/7055 (1.2%)	<0.0001	2.32	1.63-3.31	0.005	1.74	1.18-2.57
**Tobacco use**	Yes	89/10410 (0.9%)	0.77	1.04	0.79-1.37	0.21	1.20	0.90-1.60
	No	126/15576 (0.8%)		Ref.			Ref	
**Comorbidity**	Cancer							
	yes	32/1582 (2.0%)	<0.0001	3.38	2.32-4.93	0.009	1.67	1.14-2.46
	No	183/24404 (0.7%)		Ref.			Ref.	
	Diabetes							
	Yes	78/7126(1.1%)	<0.0001	1.99	1.50-2.63	0.62	0.92	0.67-1.27
	No	137/18860 (0.7%)		Ref.			Ref.	
	IHD							
	Yes	54/2298 (2.3%)	<0.0001	4.09	3.00-5.57	0.033	1.50	1.03-2.19
	No	161/ 23688 (0.7%)		Ref.			Ref.	
	CVA/TIA							
	Yes	22/1039 (2.1%)	<0.0001	3.72	2.39-5.78	0.26	1.32	0.82-2.12
	No	193/24947 (0.8%)		Ref.			Ref.	
	CHF							
	Yes	13/447 (2.9%)	<0.0001	6.46	3.68-11.35	0.071	1.80	0.95-3.39
	No	202/25539 (0.8%)		Ref.			Ref.	
	Hypertension							
	Yes	103/6936 (1.5%)	<0.0001	2.75	2.12-3.60	0.18	0.80	0.58-1.11
	No	112/19050 (0.6%)		Ref.			Ref.	
	AF							
	Yes	12/653 (1.8%)	<0.0001	3.21	1.79-5.76	0.50	0.81	0.43-1.51
	No	203/25333 (0.8%)		Ref.			Ref.	
	Vascular Disease							
	Yes	24/789 (3.0%)	<0.0001	5.48	3.58-8.39	0.011	1.83	1.15-2.92
	No	191/25197 (0.8%)		Ref.			Ref.	
	COPD							
	Yes	19/1860 (1.0%)	0.14	1.43	0.89-2.29	0.96	0.99	0.61-1.59
	No	196/24126 (0.8%)		Ref.			Ref.	
	CRF							
	Yes	12/666 (1.8%)	<0.0001	3.57	1.99-6.41	0.84	1.07	0.57-1.99
	No	203/25320 (0.8%)		Ref.			Ref.	

^a^Presented data for percentages for socioeconomic status and BMI were calculated from the total number of the available data. Missing data on socioeconomic status was 5/423 (1.2%) and for BMI was 3/527 (0.6%) for PsA+controls with VTE, respectively.

*Abbreviations*: *AF* Atrial fibrillation, *bDMARD* biologic disease-modifying anti-rheumatic drug, *BMI* body mass index, *CHF* Congestive heart failure, *CI* Confidence interval, *COPD* Chronic obstructive pulmonary disease, *CRF* Chronic renal failure, *CVA* cerebrovascular accident, *HR-* Hazard ratio, *IHD* Ischemic heart disease, *PsA* Psoriatic arthritis, *Ref.* Reference for calculation, *SD* Standard deviation, *TIA* Transient ischemic attack, *VTE* Venous thromboembolism.

Within the PsA group, only older age [p<0.0001, HR 1.08, CI (1.06-1.10)] and previous history of VTE [p<0.0001, HR 31.63, CI (14.20-70.60)] remained significantly associated with increased risk of VTE after adjusting for multiple covariates (Table 4). In our sensitivity analysis excluding PsA patients with previous VTE, older age and CHF were associated with increased risk of VTE events (Table 5). Notably, use of c/b DMARD was not associated with increased risk of VTE among PsA patients following adjustment as a time-dependent variable (Tables 4 and 5).

**Table 4:** Risk Factors for VTE among PsA Patients

			(Univariate Analysis)			Multivariate analysis		
		PsA+VTE	***P*** value	HR	95% CI	***P*** value	HR	95% CI
**Age (Mean± SD, Median)**		65.2±12.3	<0.0001	1.08	1.06-1.10	<0.0001	1.08	1.05-1.11
**sex**	Female	39/2807 (1.4%)	0.11	1.53	0.91-2.55	0.34	1.32	0.75-2.30
	Male	23/2468 (0.9%)		Ref.				
**Socio-economic status**^a^	1-low	21/1621 (1.3%)		Ref.		0.32	0.74	0.41-1.34
	2-medium	25/2043 (1.2%)	0.75	0.91	0.51-1.63	0.22	0.65	0.33-1.29
	3-high	15/1326 (1.1%)	0.61	0.84	0.43-1.63			
**BMI**^a^	BMI<25	13/1480 (0.9%)		Ref.				
	BMI≧25<30	20/1795 (1.1%)	0.67	1.16	0.58-2.34	0.46	0.77	0.37-1.57
	BMI≧30	28/1765 (.6%)	0.09	1.76	0.91-3.40	0.99	0.99	0.48-2.09
**Tobacco use**	Yes	25/2227 (1.1%)	0.82	0.94	0.57-1.56	0.90	0.97	0.56-1.66
	No	37/3048 (1.2%)		Ref.				
**Psoriasis**	Yes	52/4244 (1.2%)	0.92	1.04	0.53-2.04	0.23	0.73	0.43-1.22
	No	10/1031(0.97%)		Ref.				
**Comorbidity**	Cancer							
	Yes	8/337 (2.4%)	0.011	2.64	1.25-5.54	0.50	1.30	0.61-2.81
	No	54/4939 (1.1%)		Ref.				
	Diabetes		0.001					
	Yes	29/1781 (1.6%)		2.30	1.40-3.82	0.56	1.19	0.65-2.18
	No	33/3494 (0.9%)		Ref.				
	IHD							
	Yes	14/542 (2.6%)	0.0004	2.93	1.62-5.32	0.81	1.09	0.53-2.25
	No	48/4733 (1.0%)		Ref.				
	CVA/TIA		0.01					
	Yes	6/234 (2.5%)		2.99	1.28-6.95	0.69	1.22	0.47-3.15
	No	56/5032 (1.1%)		Ref.				
	CHF							
	Yes	4/117 (3.4%)	0.001	5.97	2.15-16.60	0.31	1.88	0.55-6.41
	No	58/5158 (1.1%)		Ref.				
	Hypertension							
	Yes	35/1589 (2.2%)	<0.0001	3.20	1.94-5.29	0.84	1.06	0.59-1.93
	No	27/3686 (0.7%)		Ref.				
	AF							
	Yes	5/159 (3.1%)	0.003	4.03	1.61-10.1	0.97	0.98	0.33-2.87
	No	57/5116 (1.1%)		Ref.				
	Vascular disease							
	Yes	5/196 (2.6%)	0.025	2.84	1.14-7.10	0.83	0.90	0.33-2.44
	No	57/5079 (1.1%)		Ref.				
	COPD							
	Yes	6/465 (1.3%)	0.55	1.30	0.56-3.00	0.77	0.88	0.37-2.08
	No	56/4810 (1.2%)		Ref.				
	CRF							
	Yes	2/159 (1.3%)	0.45	1.73	0.42-7.01	0.54	0.62	0.14-2.78
	No	60/5116 (1.2%)		Ref.				
	Past VTE							
	Yes	7/36 (18.0%)	<0.0001	31.63	14.20-70.60	<0.0001	19.60	8.37-45.94
	No	55/5236 (1.1%)		Ref.				
**Medication**	cDMARD	**Yes** 46/3705 (1.2%)		1.56	0.88-2.89	0.25	1.44	0.77-2.67
		**No** 16/1570 (1.0%)	0.12	Ref.				
	bDMARD	**Yes** 19/1989 (1.0%)	0.39	1.30	0.72-2.37	0.36	1.33	0.73-2.44
		**No** 43/3286 (1.3%)		Ref.				

^a^Presented data for percentages for socioeconomic status and BMI were calculated from the total number of the available data. Missing data for PsA+VTE group on socioeconomic status was 1/285 (0.35%) and 1/235 (0.43%), respectively.

*Abbreviations*: *AF* Atrial fibrillation, *c/b DMARD* conventional/biologic disease-modifying anti-rheumatic drugs, *BMI* body mass index, *CHF* Congestive heart failure, *COPD* Chronic obstructive pulmonary disease, *CRF* Chronic renal failure, *CVA* Cerebrovascular accident, *HR* Hazard ratio, *IHD* Ischemic heart disease, *PsA* Psoriatic arthritis, *Ref.* Reference for calculation, *SD* Standard deviation, *TIA* Transient ischemic attack, *VTE* Venous thromboembolism.

**Table 5:** Risk Factors for VTE among PsA Patients excluding cases with previous VTE

			(Univariate Analysis)			Multivariate analysis		
		PsA+VTE	***P*** value	HR	95% CI	***P*** value	HR	95% CI
**Age (Mean± SD, Median)**		65.4±12.6	<0.0001	1.08	1.06-1.11	<0.0001	1.09	1.06-1.12
**sex**	Female	35/2785 (1.3%)	0.10	1.58	0.91-2.74	0.42	1.27	0.71-2.27
	Male	20/2451 (0.8%)		Ref.				
**Socio-economic status**^a^	1-low	16/1607 (1.0%)		Ref.		0.42	0.76	0.40-1.47
	2-medium	22/2028 (1.1%)	0.91	1.04	0.55-1.98	0.56	0.81	0.39-1.66
	3-high	15/1317 (1.1%)	0.81	1.09	0.54-2.20			
**BMI**^a^	BMI<25	13/1478 (0.9%)		Ref.				
	BMI≧25<30	18/1781 (1.0%)	0.91	1.04	0.51-2.13	0.49	0.78	0.38-1.60
	BMI≧30	23/1742 (1.3%)	0.28	1.45	0.74-2.86	0.99	0.99	0.47-2.11
**Tobacco use**	Yes	21/2204 (1.0%)	0.60	0.87	0.50-1.49	0.89	0.96	0.54-1.69
	No	34/3032 (1.1%)		Ref.				
**Psoriasis**	Yes	45/4212 (1.1%)	0.72	0.88	0.44-1.75	0.54	0.80	0.39-1.63
	No	10/1024 (1.0%)		Ref.				
**Comorbidity**	Cancer							
	Yes	8/332 (2.4%)	0.003	3.07	1.45-6.50	0.19	1.68	0.77-3.65
	No	47/4904 (1.0%)		Ref.				
	Diabetes							
	Yes	23/1753 (1.3%)	0.375	0.75	0.40-1.41	0.72	1.13	0.59-2.13
	No	32/3483 (0.9%)		Ref.				
	IHD							
	Yes	11/535 (2.1%)	0.006	2.55	1.32-4.94	0.85	0.93	0.43-2.00
	No	44/4701 (0.9%)		Ref.				
	CVA/TIA							
	Yes	6/237 (2.5%)	0.003	3.55	1. 52-8.31	0.21	1.79	0.72-4.46
	No	49/4999 (1.0%)		Ref.				
	CHF							
	Yes	4/116 (3.4%)	<0.0001	7.22	2.58-20.2	0.03	3.84	1.16-12.6
	No	51/5120 (1.0%)		Ref.				
	Hypertension							
	Yes	29/1565 (1.9%)	<0.0001	2.78	1.64-4.73	0.87	1.05	0.57-1.94
	No	26/3671(0.7%)		Ref.				
	AF							
	Yes	2/153 (1.3%)	0.41	1.81	0.44-7.44	0.24	0.41	0.089-1.84
	No	53/5083 (1.0%)		Ref.				
	Vascular disease							
	Yes	4/192 (2.1%)	0.064	2.62				
	No	51/5044 (1.0%)		Ref.	0.94-7.25	0.74	0.83	0.28-2.47
	COPD		0.94					
	Yes	4/460 (0.9%)		0.96	0.35-2.67	0.51	0.71	0.25-1.99
	No	51/4776 (1.1%)		Ref.				
	CRF							
	Yes	1/157 (0.6%)	1.00	1.00	0.14-7.27	0.20	0.26	0.032-2.02
	No	54/5079 (1.1%)		Ref.				
**Medication**	cDMARD	**Yes** 42/3681 (1.1%)		1.49				
		**No** 13/1555 (0.8%)	0.22	Ref.	0.79-2.82	0.18	1.58	0.81-3.10
	bDMARD	**Yes** 19/1974 (1.0%)	0.94	1.02	0.57-1.85	0.15	1.58	0.85-2.92
		**No** 36/3262 (1.1%)		Ref.				

^a^Presented data for percentages for socioeconomic status and BMI were calculated from the total number of the available data. Missing data for PsA+VTE group on socioeconomic status was 2/284 (0.70%) and 1/235 (0.43%), respectively.

*Abbreviations*: *AF* Atrial fibrillation, *c/b DMARD* conventional/biologic disease-modifying anti-rheumatic drugs, *BMI* body mass index, *CHF* Congestive heart failure, *COPD* Chronic obstructive pulmonary disease, *CRF* Chronic renal failure, *CVA* Cerebrovascular accident, *HR* Hazard ratio, *IHD* Ischemic heart disease, *PsA* Psoriatic arthritis, *Ref.* Reference for calculation, *SD* Standard deviation, *TIA* Transient ischemic attack, *VTE* Venous thromboembolism.

## Discussion

In our study, a large, retrospective population study reporting on risk factors associated with VTE among PsA patients in real life, we found no increased risk of VTE occurrence among PsA patients compared to controls in the general population following adjustment for multiple covariates including associated comorbidities. However, we did observe that the risk of VTE was elevated by 40% in PsA patients compared to controls from the general population matched by age, sex, and ethnicity in our univariate analysis, suggesting that underlying comorbidities may play a significant role in increasing the risk of VTE among PsA patients. Therefore, screening for the presence of comorbidities, especially cardiovascular risk factors and previous history of VTE, are important in the treatment management and may influence medication choices.

In our review of the literature, the consensus of research ascertains that there is an increased risk of VTE in PsO [6, 12] with a recent meta-analysis [4] showing an increased relative risk of VTE occurrence of 1.46 in PsO. Notably, the study by Ogdie et al. [12] even demonstrates a correlation between increasing VTE risk and increasing severity of PsO, where HR of VTE events increases from 1.35 in mild psoriasis to 2.06 in severe psoriasis.

In contrast, the risk of occurrence of VTE events in patients with PsA has only been examined by a small number of studies. One such study is a population-based cohort study conducted in 2017, set out to identify, assess, and compare risk of cardiovascular events among patients diagnosed with PsA, ankylosing spondylitis (AS), and undifferentiated spondyloarthritis in the Swedish National Patient Register compared to the general population in the Swedish Population Register [13]. The adjusted prevalence ratios of cardiovascular events including VTE were higher in all 3 patient cohorts in comparison to the general population, with an increase in HR for VTE by approximately 50% in all 3 patient cohorts [13]. Of note, this increase in VTE risk was not noted in male PsA patients.

Another population-based study was conducted in the United Kingdom to assess for any increase in risk of VTE among patients with rheumatoid arthritis (RA), PsO and PsA relative to the general population [12]. This study found that patients with RA (with and without a DMARD prescription) and patients with mild PsO had elevated risks of VTE (HR 1.35, 1.29, and 1.07, respectively) even after adjusting for traditional risk factors. Severe PsO patients and PsA patients prescribed a DMARD had an elevated but not statistically significant risk of VTE, with similar findings for DVT. The age-and-sex-adjusted risk of PE was elevated in RA, severe PsO as well as in PsA patients prescribed a DMARD [12].

The discrepancy between our findings and the findings in these studies may stem from differences among the study populations and variability in study designs. For instance, in the study by Ogdie et al. [12], a subclassification of VTE events into DVT and PE as secondary outcomes gave divergent results regarding VTE risk. Moreover, patients diagnosed with both PsO and PsA were studied as two separate patient cohorts with separate matched controls, whereas we included in our study patients with PsA in a single cohort regardless of presence of PsO, and evaluated PsO as a covariate which was not found to increase risk of VTE within the PsA group. Moreover, in the study by Ogdie et al., covariates were selected using a purposeful selection modeling approach as part of the study design, while we used forward selection model in multivariate analysis [12].

Aside from the aforementioned studies, a very recent study by Damian et al. from 2021 [14] examined the incidence rates of DVT, PE and VTE events among patients with psoriatic disease (PsD), demonstrating a 4.6% cumulative incidence rate of VTE by age 80; and noting older age, diabetes, and corticosteroid usage as independent risk factors for VTE events. Unlike our study, this study examined PsD in the analysis of VTE incidence, and since it did not include a comparison group, no conclusion can be drawn regarding relative risk of VTE in PsA patients from this study.

Similar to other studies, [15] including this latest study by Damian et al. [14], we found that older age was a significant risk factor for occurrence of VTE events among PsA patients, as was previous history of VTE [16]. Interestingly, tobacco use, which has been shown by several studies to increase risk of VTE, [17, 18] was not associated with higher risk of VTE among PsA patients in our cohort (Table 4).

We did not find an association between VTE risk with either cDMARD or bDMARD use even following adjustment for time-dependency. It is also equally important to note, however, that as our study was conducted prior to the advent of Janus kinase (JAK) inhibitors as part of the PsA treatment arsenal, these medications were excluded from our analysis. This is of significance as JAK inhibitors have subsequently been associated with an increase in risk of VTE events in patients with inflammatory arthritis and cardiovascular comorbidities [7] and thus may affect medication choices in these patients.

In regards to potential underlying mechanisms for VTE occurrence among patients with PsD, there are suggestions that inflammatory states can induce atherosclerosis and atherothrombosis, leading to the development of cardiovascular events [6, 18–20]. In the case of VTE events, inflammation-induced venous thrombosis may develop even in the absence of vessel wall damage, with the coagulation cascade causing a vicious cycle by further augmenting inflammation via thrombin-induced secretion of pro-inflammatory cytokines and growth factors, and via platelet-induced activation of dendritic cells [21]. Accordingly, in our study and in accordance with other studies, [1, 22] we found an increase in prevalence of morbid obesity among PsA patients, with accumulating data supporting the concept that obesity is a pro-inflammatory state caused by the endocrine and metabolic activity of the adipose tissue [23], thus increasing the risk of VTE [16, 18]. Other traditional risk factors for VTE, such as IHD and vascular disease, which are also associated with underlying inflammation, [24] were also found to be more prevalent among individuals with VTE in our study.

Notably, recent research suggests that cardiovascular morbidity among PsA patients involves a complex interplay between discrete and unique inflammatory and noninflammatory factors inherent to PsA itself, such as angiogenesis, oxidative stress and endothelial dysfunction [24–26], which can be found in PsA patients even with no classic CVD risk factors [26–28]. Supporting this concept, for instance, are recent studies showing that severe cases of PsO and PsA promote platelet aggregation and activation, factors related to atherothrombosis and VTE [29–31]. This evidence points to an inherent thrombotic risk in PsA, making VTE surveillance important in this patient population.

Possible limitations in our study include the relatively small number of VTE events among PsA patients captured in the CHS database, which thus limits the power to detect an association between PsA and VTE occurrence in the multivariable analysis.

Additional general limitations of administrative database research include lack of clinical data on PsA disease activity, the potential presence of unmeasured confounders, and the potential misclassification of cases/controls and VTE events including lack of subclassification of VTE events into DVT and PE in this study. Our study focused on PsA and did not analyze PsO alone. Despite the aforementioned limitations, our study is founded on a database of 4.7 million individuals featuring data on the long-term follow-up of PsA patients in real life.

## Conclusion

In conclusion, our study suggests that the increased risk of VTE in PsA patients appears to be related to the underlying comorbidities and not independently associated with PsA. Among PsA patients, older age and previous history of VTE were associated with increased risk of VTE. Given the association of VTE with comorbidities common in PsA patients, our findings do support continued research into VTE risk factors in PsA and active surveillance of this patient population for VTE occurrence especially in an era of medications such as JAK inhibitors.

## Data Availability

The datasets used and/or analyzed during the present study are available from the corresponding author on reasonable request.

## Abbreviations

AS: Ankylosing spondylitis
BMI: Body mass index
CHF: Congestive heart failure
CHS: Clalit health services
CI: Confidence interval
CVD: Cardiovascular disease
c/b DMARDs: Conventional and biologic disease-modifying anti-rheumatic drugs
DVT: Deep venous thrombosis
HR: Hazard ratio
IHD: Ischemic heart disease
JAK inhibitors: Janus kinase inhibitors
PE: Pulmonary embolism
PsA: Psoriatic arthritis
PsD: Psoriatic disease
PsO: Psoriasis
PVD: Peripheral vascular disease
RA: Rheumatoid arthritis
SD: Standard deviation
SES: Socioeconomic status
VTE: Venous thromboembolic events
